# Clinical spectrum and genetic landscape for hereditary spastic paraplegias in China

**DOI:** 10.1186/s13024-018-0269-1

**Published:** 2018-07-06

**Authors:** En-Lin Dong, Chong Wang, Shuang Wu, Ying-Qian Lu, Xiao-Hong Lin, Hui-Zhen Su, Miao Zhao, Jin He, Li-Xiang Ma, Ning Wang, Wan-Jin Chen, Xiang Lin

**Affiliations:** 1 Department of Neurology and Institute of Neurology, The First Affiliated Hospital of Fujian Medical University, Fuzhou, 350005 China; 2 Department of Anatomy, Histology and Embryology, Shanghai Medical College, Fudan University, Shanghai, 200032 China; 3 Fujian Key Laboratory of Molecular Neurology, Fujian Medical University, Fuzhou, 350005 China

**Keywords:** Hereditary spastic paraplegias, Clinical features, Mutational spectrum, Heterogeneity, Founder effect, Mitochondria, Autolysosomes

## Abstract

**Background:**

Hereditary spastic paraplegias (HSP) is a heterogeneous group of rare neurodegenerative disorders affecting the corticospinal tracts. To date, more than 78 HSP loci have been mapped to cause HSP. However, both the clinical and mutational spectrum of Chinese patients with HSP remained unclear. In this study, we aim to perform a comprehensive analysis of clinical phenotypes and genetic distributions in a large cohort of Chinese HSP patients, and to elucidate the primary pathogenesis in this population.

**Methods:**

We firstly performed next-generation sequencing targeting 149 genes correlated with HSP in 99 index cases of our cohort. Multiplex ligation-dependent probe amplification testing was further carried out among those patients without known disease-causing gene mutations. We simultaneously performed a retrospective study on the reported patients exhibiting HSP in other Chinese cohorts. All clinical and molecular characterization from above two groups of Chinese HSP patients were analyzed and summarized. Eventually, we further validated the cellular changes in fibroblasts of two major spastic paraplegia (SPG) patients (SPG4 and SPG11) in vitro.

**Results:**

Most patients of ADHSP (94%) are pure forms, whereas most patients of ARHSP (78%) tend to be complicated forms. In ADHSP, we found that SPG4 (79%) was the most prevalent, followed by SPG3A (11%), SPG6 (4%) and SPG33 (2%). Subtle mutations were the common genetic cause for SPG4 patients and most of them located in AAA cassette domain of spastin protein. In ARHSP, the most common subtype was SPG11 (53%), followed by SPG5 (32%), SPG35 (6%) and SPG46 (3%). Moreover, haplotype analysis showed a unique haplotype was shared in 14 families carrying c.334C > T (p.R112^*^) mutation in *CYP7B1* gene, suggesting the founder effect. Functionally, we observed significantly different patterns of mitochondrial dynamics and network, decreased mitochondrial membrane potential (Δψm), increased reactive oxygen species and reduced ATP content in SPG4 fibroblasts. Moreover, we also found the enlargement of LAMP1-positive organelles and abnormal accumulation of autolysosomes in SPG11 fibroblasts.

**Conclusions:**

Our study present a comprehensive clinical spectrum and genetic landscape for HSP in China. We have also provided additional evidences for mitochondrial and autolysosomal-mediated pathways in the pathogenesis of HSP.

**Electronic supplementary material:**

The online version of this article (10.1186/s13024-018-0269-1) contains supplementary material, which is available to authorized users.

## Background

Hereditary spastic paraplegias (HSP) refer to a heterogeneous group of rare neurodegenerative disorders. HSP have a variable age at onset (AAO) and are mainly characterized by slow progressive bilateral spasticity and weakness of lower limbs [1]. The estimated prevalence of HSP is about 2–10/100000 in the general population [2]. Clinically, HSP cases are divided into pure and complicated forms. Pure form is characterized by pyramidal signs, associated with isolated spasticity and weakness confined to the lower limbs [3]. These patients rarely need wheel chairs but may use crutches during the disease course, even they usually have normal lifespan [4]. However, additional severe neurological symptoms can be exhibited in the complicated form, such as ataxia, dysarthria, cognitive impairment, mental retardation, epilepsy, peripheral neuropathy, as well as extra-neurological signs like ophthalmoplegia [5]. The functional handicap and lifespan are associated with the full clinical manifestation in complicated form [6].

Genetically, HSP are highly heterogeneous, with 78 spastic paraplegia (SPG) associated loci and more than 60 identified genes [5, 7]. Moreover, at least 20 additional genes have recently been correlated to HSP [5, 6]. HSP can be transmitted with all classical modes of inheritance, including autosomal dominant (AD), autosomal recessive (AR), X-linked or mitochondrial maternal transmission. Autosomal dominant hereditary spastic paraplegia (ADHSP) is the most prevalent form of HSP and accounts for approximately 70% of cases [8]. SPG4 (50%), SPG3A (10%), SPG31 (4.5%) and SPG10 (2.5%) are the most common causes of ADHSP, in decreasing order of prevalence [6]. Autosomal recessive HSP (ARHSP) is the next largest group of HSP. Mutations in the *CYP7B1* (SPG5), *SPG7* (SPG7), *SPG11* (SPG11) and *ZFYVE26* (SPG15) gene are described as the most frequent causes for ARHSP [9, 10]. However, their relative frequencies vary largely depending on the distinct geographical origin.

Membrane trafficking and axonal transport are emerging as potentially main scenario for HSP [4]. The considerable known HSP causative genes encode proteins mainly involved in microtubule dynamics, endoplasmic reticulum morphogenesis, vesicular formation, lipid metabolism and endosomal functions [3, 11]. However, growing evidences revealed that above theories cannot fully explain the etiology of HSP [12]. Identifying the role of responsible genes could have far-reaching effects on patients, as it would reveal the cause of the disease, and help to better understand the correlation between genotypes and phenotypes. With the growing number of causative genes and the complexity of genotypes and phenotypes, it is difficult to achieve a definite genetic diagnosis in many affected individuals using traditional sanger sequencing. To date, comprehensive review of clinical spectrum and genetic landscape in a large cohort of HSP patients have not yet been presented in China.

In the present study, we firstly investigated the causative genes in 99 both ADHSP and ARHSP families in our cohort using the targeted next generation sequencing (NGS) or multiplex ligation-dependent probe amplification (MLPA) approach. We then retrospectively analyzed and summarized the clinical spectrum and genetic landscape in 47 documented families of our cohort, and in 140 confirmed HSP families of other reported cohorts in China. During the course of these studies, we discovered that SPG4 (79%), SPG3A (11%), SPG6 (4%) and SPG33 (2%) were the most frequently found in Chinese ADHSP patients, and were mainly pure forms with wide range AAO. Meanwhile, Chinese ARHSP patients were more complex in clinical terms with early AAO. The major subtypes of ARHSP in decrease order of frequency were SPG11 (53%), SPG5 (32%), SPG35 (6%) and SPG46 (3%). Moreover, we determined that the high prevalence of SPG5 resulted from the founder effect. On these basis, we further conducted functional studies on two major subtypes, SPG4 and SPG11, patients derived fibroblasts to elucidate the pathogenic mechanisms. Overall, these results have provided a new perspective to assess the clinical features, genetic distribution and physiopathology for Chinses HSP patients.

## Methods

### Subjects recruitment

Subjects participated in our cohort were recruited from the Department of Neurology, First Affiliated Hospital of Fujian Medical University. In total, 99 families fulfilling the clinical diagnostic criteria for HSP were enrolled irrespective of their genetic diagnosis [13]. Patients in other cohort were collected by review of 41 reported studies exhibiting HSP in China (Additional file 1: Table S1). A total of 418 HSP patients from 140 families were included.

In addition, 200 unrelated Chinese individuals without history of spastic paraplegia were selected as the control group.

### Targeted NGS and data analysis

Genomic DNA was extracted from peripheral vein blood using a QIAamp DNA Blood Mini Kit (Qiagen, Hilden, Germany) according to the manufacturer’s instructions. Targeted NGS panel was designed to cover 149 genes known to be correlated with HSP (Additional file 1: Table S2). The whole exons, along with 20 base pairs of flanking sequences, of targeted genes were enriched with the TruSeq DNA LT Sample Prep Kit v2 (Illumina) according to the manufacturer’s standard protocol. DNA libraries were subjected to paired-end sequencing on the Illumina High Seq 3000 platform. After eliminating the low-quality FASTQ sequencing reads by Trim Galore, we mapped the remaining high-quality reads to the human reference genome (UCSC hg19) with Burrows-Wheeler Aligner. The variants were called using Genome Analysis Tool Kit, and were annotated with the annotate variation.

All the variants were further filtered by 1000 Genomes Project (http://phase3browser.1000genomes.org/index.html), Exome Sequencing Project (http://evs.gs.washington.edu/EVS/) and Exome Aggregation Consortium database (http://exac.broadinstitute.org/). The property of variants, either benign or pathogenic, were further analyzed by SIFT (http://sift.jcvi.org), PolyPhen-2 (http://genetics.bwh.harvard.edu/pph2/), and Mutation Taster (http://www.mutationtaster.org). Finally, the pathogenic mutations were compared with the Human Gene Mutation Database (http://www.hgmd.cf.ac.uk/) to determine whether they were known or novel mutations.

### Sanger sequencing

Specific primers for amplification of mutations were designed by the Primer 5 software listed in Additional file 1: Table S3. The targeted regions were amplified with polymerase chain reaction (PCR) in a 2720 Thermal Cycler (ABI). After purification, the amplified products were electrophoresed on an ABI 3730XL Automated DNA analyser (PE Applied Biosystems, Foster City, CA) according to standard protocols. The sequencing results were assembled with chromas software.

### MLPA testing

MLPA testing was performed in the patients without known disease-causing gene mutations for detection of large deletions or copy number variations. The MLPA analyses were performed according to the manufacturer’s protocol using the following SALSA MLPA probemixes: P165-C2 (coverage: *ATL1*, *SPAST*), P211-B4 (coverage: *SPAST*, *NIPA1*), P306-B1 (coverage: *SPG11*) and P213-B2 (coverage: *REEP1*, *SPG7*) (MRC-Holland, Amsterdam, the Netherlands). Data were collected by Genemapper 3.0 (Applied Biosystems, California, USA) and analyzed using Coffalyser software.

### Haplotype analysis

Based on the database of Southern Han Chinese in Haplotype Map Project (https://www.ncbi.nlm.nih.gov/variation/tools/1000genomes/), 9 tagSNPs (rs9298109, rs6985116, rs7842714, rs116843046, rs200737038, rs4465006, rs3779869, rs8192906 and rs4367588) were selected for further haplotype analysis of unrelated SPG5 families. These tagSNPs are linkage to *CYP7B1* gene and spanning ~ 1.5-Mb interval. PCR and sanger sequencing were used to identify the specific genotype of tagSNP. Primers for amplification were listed in Additional file 1: Table S3.

### Study on the mutational function in vitro

#### DNA constructs and cell transfection

The human M1- and M87-spastin cDNAs was prepared as described previously [14]. Briefly, wild-type spastin cDNAs was cloned into the *BamH*I and *Not*I site of the eukaryotic expression vector of pCMV6-Entry, with HA tag at the N terminus. To generate mutagenesis plasmid, the Mut Express II Fast Mutagenesis Kit V2 (Vazyme) was used according to the manufacturer’s instructions. The construction was verified by DNA sequencing after ligation, transformation and extraction. The primers for plasmid construct were summarized in Additional file 1: Table S3.

HEK293T cells were cultured in Dulbecco’s modified Eagle’s medium (DMEM) supplemented with 10% fetal bovine serum (FBS) and maintained at 37 °C under 5% CO_2_/air. Transient transfections were performed using Lipofectamine 3000 (Thermo Scientific) for plasmid DNAs.

#### Primary fibroblasts culture and treatment

Primary fibroblast cell lines were established from skin biopsies with punches (Electron microscopy sciences), obtained from the probands of family F12 of SPG4 and family F4 of SPG11. Fibroblasts were maintained in DMEM supplemented with 20% FBS and 1% penicillin/ streptomycin.

Autophagy was induced by amino acid and serum starvation in Earle’s Balanced Salt Solution (Thermo Scientific) for 6 h.

#### Reverse transcription polymerase chain reaction (RT-PCR) and quantification PCR (qPCR)

Total RNA from leukocytes or fibroblasts were extracted with the TRIzol kit (Invitrogen) according to the manufacturer’s instructions. A 0.5-μg aliquot of total RNA was reversely transcribed into cDNA.

RT-PCR was performed in a 2720 Thermal Cycler (ABI), and the resulting products were visualized on 2.5% agarose gels under UV transilluminator (Tanon, China). QPCR were performed with SYBR@Premix EX TaqTM II kit (Takara) and run on an Applied Biosystems Stepone Real-time PCR System. The primers for amplification are listed in Additional file 1: Table S3.

#### Detection of mitochondrial membrane potential (MMP, Δψm) by JC-1

Fibroblast cells were cultured on glass slide. JC-1 dye solution was added to cells in medium and incubated for 20 min at 37°C (Addition file 1: Table S4), according to the manufacture’s recommended protocol. After washing with cold incubation buffer solution, JC-1 staining of mitochondria in live cells was immediately observed using a Leica TCS SP8 confocal system. The wavelength at excitation/emission 525/590 nm was used to assess JC-1 aggregates, and at excitation/emission 488/525 nm was used to assess JC-1 monomer.

#### Detection of intracellular reactive oxygen species level by DCFH-DA

Reactive oxygen species (ROS) in fibroblasts were detected using a fluorescent probe, 2′7’-dichlorofluorescein diacetate (DCFH-DA). DCFH-DA is hydrolyzed to DCFH, which further be oxidized to fluorescent DCF by intracellular ROS. Briefly, fibroblasts seeded on glass slide were rinsed with 1 × PBS and incubated with DCFH-DA for 20 min at 37°C (Addition file 1: Table S4), according to the manufacture’s recommended protocol. After washing with 1 × PBS, DCFH-DA labeled mitochondria in live cells were immediately observed using a Leica TCS SP8 confocal system. The wavelength at 488 nm excitation and 525 nm emission was used to assess DCF.

#### Measurement of ATP content

ATP content was determined in fibroblasts cell lysates using an ATP bioluminescence assay kit (Addition file 1: Table S4), according to the manufacture’s recommended protocol. Measurements were performed in a luminometer (Tecan, Austria). The relative ATP content was recorded as ATP value (nmol) /protein value (mg) ratio.

#### Immunocytochemistry (ICH) and quantification

The fibroblasts were fixed with 4% paraformaldehyde (10–20 min at room temperature), rinsed three times with 1× PBS (Medicago AB) and incubated in a blocking buffer (10% donkey serum and 0.2% triton X-100 in PBS) for 1 h and then incubated with primary antibodies (Additional file 1: Table S4) overnight in a refrigerator at 4 °C. Fluorescence conjugated secondary antibodies (Thermo Scientific) and DAPI (Sigma) were used at 1:1000 dilution. Images were captured by a Leica TCS SP8 confocal system.

Image-J software (NIH, MD, USA) was used for further quantification of the cell population, particle number, lysosome diameter and fluorescence intensity. Quantification was performed by a person blind to the experiment and replicated in four random visual fields from three independent experiments.

#### Western blotting

The cells were harvested in the cell lysis reagent (Sigma) supplemented with a 1% protease inhibitor PMSF (Beyotime) and centrifuged at 13,000 rpm for 20 min at 4 °C. Protein extracts were separated on 12% SDS-PAGE gels and immunoblotted with primary antibodies listed in Additional file 1: Table S4. HRP-conjugated secondary antibodies (1:5000, Santa Cruz) were used to detect primary antibodies and proteins were visualized by chemiluminescence (Millipore).

#### Statistical analysis

All data were presented as the mean ± SEM. SPSS 16.0 was used for statistical comparisons, and significance was determined using the Student’s *t*-test. Differences were considered statistically significant when the *P* value was less than 0.05 (^*^).

## Results

NGS targeting 149 genes correlated with HSP was performed in all probands of our cohort. In aggregate, approximately 99.9% target bases were covered by the probe and over 82.3% of bases were covered at greater than 50×, suggesting an accepted threshold for variant calling. After filtering the sequence data down to nonsynonymous or loss-of-function (LOF) variants with allele frequencies less than 1% in the 1000 Genomes, ESP5400 and ExAC, we finally identified 37 novel or known pathogenic variants in both 22 ADHSP and 25 ARHSP families. All these mutations have been confirmed by sanger sequencing and were not detected in 200 ethnically matched controls. Moreover, exon deletion of *SPAST* gene was detected among three SPG4 families by MLPA. With retrospective analysis, 89 ADHSP and 51 ARHSP families in other Chinese cohorts were also included in the present study (Additional file 1: Table S1).

**Fig. 1:**
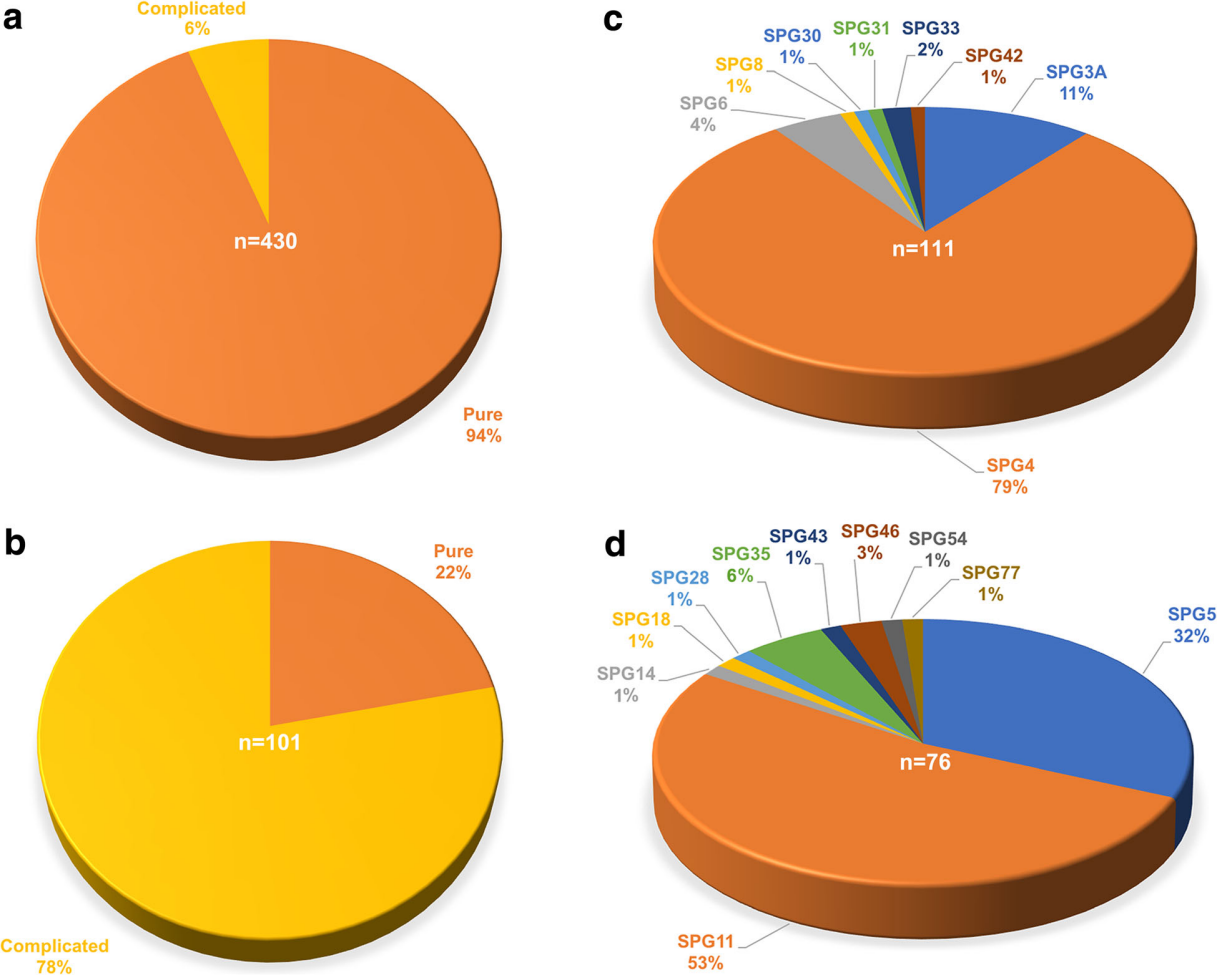
Distribution of clinical features and identified genetic subtypes of HSP in China. **a** Distribution of pure and complicated forms in ADHSP patients (*n* = 430). **b** Distribution of pure and complicated forms in ARHSP patients (*n* = 101). **c** Distribution of genetic cause in ADHSP families (*n* = 111). **d** Distribution of genetic cause in ARHSP families (*n* = 76)

Collectively, a considerable number of ADHSP patients (94%) were pure forms, whereas most ARHSP patients (78%) tended to be complicated forms (Fig. 1a and b). In ADHSP, we found that SPG4 was the most frequent subtype, account for 79% of all cases with causal dominant genes in China (Fig. 1c). SPG4 was followed by SPG3A (11%), SPG6 (4%) and SPG33 (2%). In ARHSP, the most common subtype was SPG11 (53%), followed by SPG5 (32%), SPG35 (6%) and SPG46 (3%) (Fig. 1d). Importantly, some rare subtypes of our cohort patients including SPG8 (1%), SPG28 (1%), SPG30 (1%), SPG43 (1%) and SPG54 (1%) were firstly presented in China.

### The clinical features and mutational spectrum in Chinese ADHSP patients

#### Mutations in *SPAST* gene cause ADHSP

SPG4 is caused by mutations in *SPAST* gene [15]. The clinical features of Chinese SPG4 patients were summarized in Additional file 1: Table S5. The mean ± SD AAO in these patients was 25.01 ± 15.84 years, ranging from 1 to 62 years old. The mean ± SD disease duration was 17.91 ± 14.98 years. Most cases (93.8%) had the core features of HSP, and were considered as prototypical pure forms. Moreover, peripheral neuropathy was the most common symptom in complicated patients.

**Fig. 2:**
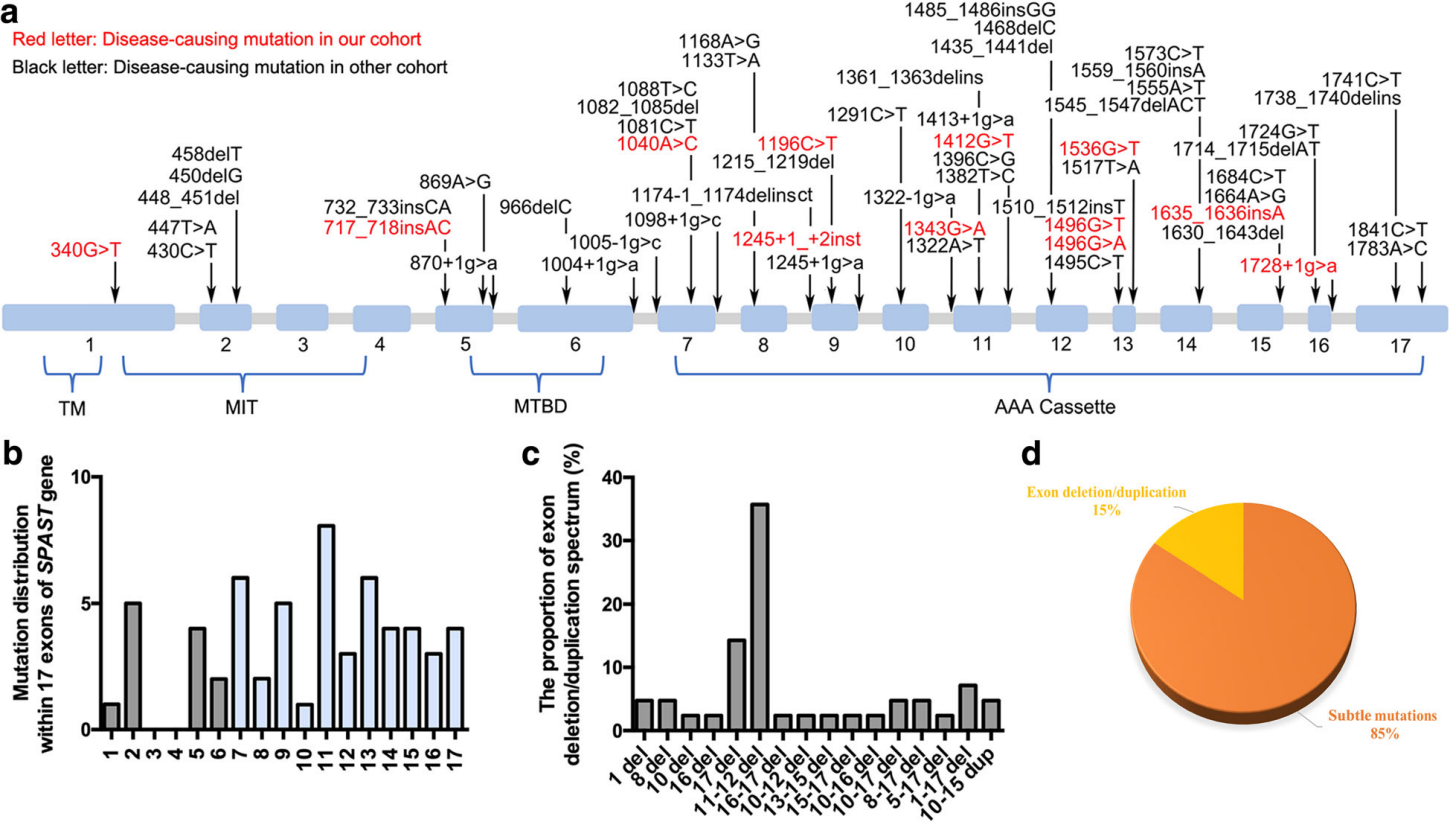
Mutational spectrum of *SPAST* gene in Chinese SPG4 patients. **a** Schematic representation of the mutational location in cDNA of *SPAST* gene detected in Chinese SPG4 patients (*n* = 272). The *SPAST* gene spans the region of ~ 90 kb of genomic DNA and contains 17 exons. Mutations detected in the current study are indicated in red characters. TM (57–79 amino acids), MIT (116–197 amino acids), MTBD (270–328 amino acids) and AAA cassette (342-599  amino acids) are highlighted. **b** Distribution of mutations are shown within 17 exons of *SPAST* gene. **c** The proportion of exon deletion and duplication spectrum. **d** The percentage of patients who carried micro mutations or exon deletion/duplication in *SPAST* gene

Spastin, encoded by *SPAST* gene, contains four functional domains including transmembrane (TM) domain, microtubule interacting and trafficking (MIT) domain, microtubule-binding domain (MTBD), and AAA (*A*TPases *a*ssociated with diverse cellular *a*ctivities) cassette domain [16]. To date, 46 subtle mutations in *SPAST* gene have been reported in Chinese SPG4 patients (Additional file 1: Table S1). In our cohort, 16 SPG4 families were identified to carry 14 pathogenetic mutations: seven were missense mutations, two were frameshift mutations, one was stop-gain mutation, two were splicing mutations and two were exons deletion (Additional file 2: Figure S1). All of these 58 subtle mutations widely distributed in four domains of spastin protein and mainly located in the AAA cassette (Fig. 2a and b), accounting for up to 78.3%. Except for subtle mutations, 16 kinds of large deletions or duplications of *SPAST* gene have also been detected, of which the exon 11–12 deletion was the most common mode (Fig. 2c). By comparing the frequency, we further discovered that subtle mutations were the much more common (85%) genetic cause for SPG4 (Fig. 2d).

**Fig. 3:**
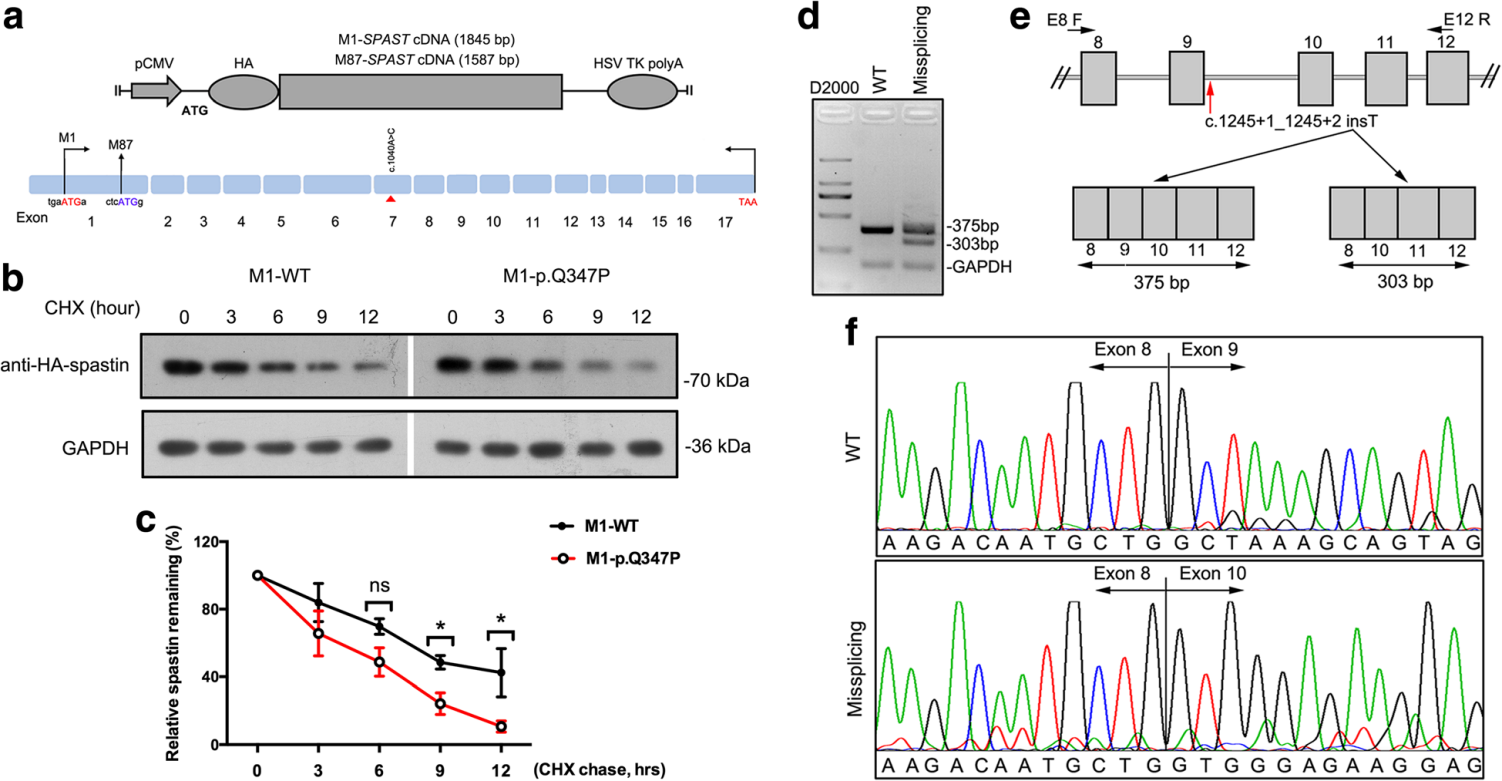
Functional analysis of novel mutations in *SPAST* gene. **a** Schematic of the human M1- and M87-spastin expression vectors for missense mutation c.1040A > C (p.Q347P). The weak Kozak sequence tgaATGa is present at the M1 initiation codon. A better Kozak sequence ctcATGg is present at the M87 initiation codon. **b** Time-course stability analysis of mutant spastin (M1-p.Q347P) by western blot . Cells were collected at 0, 3, 6, 9, 12 h following treatment with CHX. **c** Statistical analysis of b (*n* = 3). All values are normalized to untreated controls. **d** RT-PCR analysis of mutation c.1245 + 1_c.1245 + 2 insT. Two bands (size in 375 bp and 303 bp) were observed in the missplicing mutation. **e** Schematic diagram showing the skipping of exon 9 for c.1245 + 1_c.1245 + 2 insT mutation (red arrow indicated). **f** Sanger sequencing confirmed that the skipping of exon 9 had occurred. Error bars indicate SEM. *, *P* < 0.05. ns, not significant (*P* > 0.05)

Four novel mutations, c.717_718insAC (p.S243Lfs^*^12), c.1040A > C (p.Q347P), c.1245 + 1_c.1245 + 2insT and c.1635_1636insA (p.G546Rfs^*^3) were identified in our cohort. Based on the standards and guidelines for the interpretation of sequence variants [17], p.S243Lfs^*^12 and p.G546Rfs^*^3 were considered as both LOF mutations (Additional file 2: Figure S2), which was the very strong evidence for pathogenic effect. To further confirm the pathogenesis of c.1040A > C and c.1245 + 1_c.1245 + 2insT, we studied the functional effect on spastin. As a result of two different initiation codons, two primary isoforms of spastin were synthesized: a full-length isoform called M1 and a slightly shorter isoform called M87. M87 was detectably presented in all tissues, whereas M1 was only expressed in adult spinal cord [12]. Therefore, we introduced the p.Q347P missense mutation into M1- and M87-spastin expression HA-tag vectors and overexpressed them in HEK 293 T cell (Fig. 3a). Immunoblotting of HA showed that spastin level of p.Q347P was found to be comparable to that of WT (Additional file 2: Figure S2). To further study its influence on stability of spastin, we performed cycloheximide (CHX, 40 μg/ml) chase assay to inhibit de-novo protein synthesis and reassessed the protein expression levels after different treatment durations. The treatment of CHX resulted in rapid degradation of p.Q347P protein (Fig. 3b and c; Additional file 2: Figure S2), indicating that the missense mutation affected protein half-life of spastin. In addition, we also validated the effect of splicing mutation c.1245 + 1_c.1245 + 2insT on patient’s leukocytes derived mRNA. A different product in size from the RT-PCR of *SPAST* cDNA from the patient was detected (Fig. 3d), indicating the presence of an aberrant splice event (Fig. 3e). The sequencing of amplification products confirmed that alternative splicing result in the skipping of exon 9 in mutant transcript (Fig. 3f).

#### Mutations in *ATL1*, *KIAA0196*, *KIF1A* and *ZFYVE27* gene cause ADHSP

Mutations in *KIAA0196* (SPG8), *KIF1A* (SPG30) and *ZFYVE27* (SPG33) gene of our cohort ADHSP patients were firstly presented in China. Three missense mutations and one frameshift mutation of these genes (p.T845A in *KIAA0196*, p.I37T in *KIF1A*, p. N88Rfs^*^7 and p.A290T in *ZFYVE27*) were detected in four ADHSP families (Additional file 2: Figure S3). Patients carrying p.T845A in *KIAA0196* and p.A290T in *ZFYVE27,* were classified as complicated HSP, whereas others presented as pure form. Structural magnetic resonance images (MRI) of the complicated patient carrying p.A290T revealed agenesis of corpus callosum and leukoencephalopathy. In addition, we also identified two missense mutations (p.A350S and p.R416C) of *ATL1* (SPG3A) in one family with pure form and another family with complicated form, respectively (Additional file 2: Figure S3). Both probands with *ATL1* mutations had a very young AAO. Intriguingly, the mother of the proband carrying the reported mutation p.R416C, remained asymptomatic presently.

### The clinical features and mutational spectrum in Chinese ARHSP

#### Mutations in *SPG11* gene cause ARHSP

SPG11 is caused by mutations in *SPG11* gene [18]. The clinical characterization of Chinese SPG11 patients were summarized in Additional file 1: Table S6. In general, SPG11 patients had early AAO (12.71 ± 4.48 years, mean ± SD), ranging from 4 to 20 years old. The mean ± SD disease duration was 12.46 ± 8.43 years. The majority of patients (78.5%) initially presented with gait disturbance. Except for the typical manifestation of HSP, most cases combined with cognitive deficit (78.9%), dysarthria (73.1%), mental retardation (53.9%) and peripheral neuropathy (61.6%). Notably, 92.3% of SPG11 patients presented a typical sign of thin corpus callosum on MRI.

**Fig. 4:**
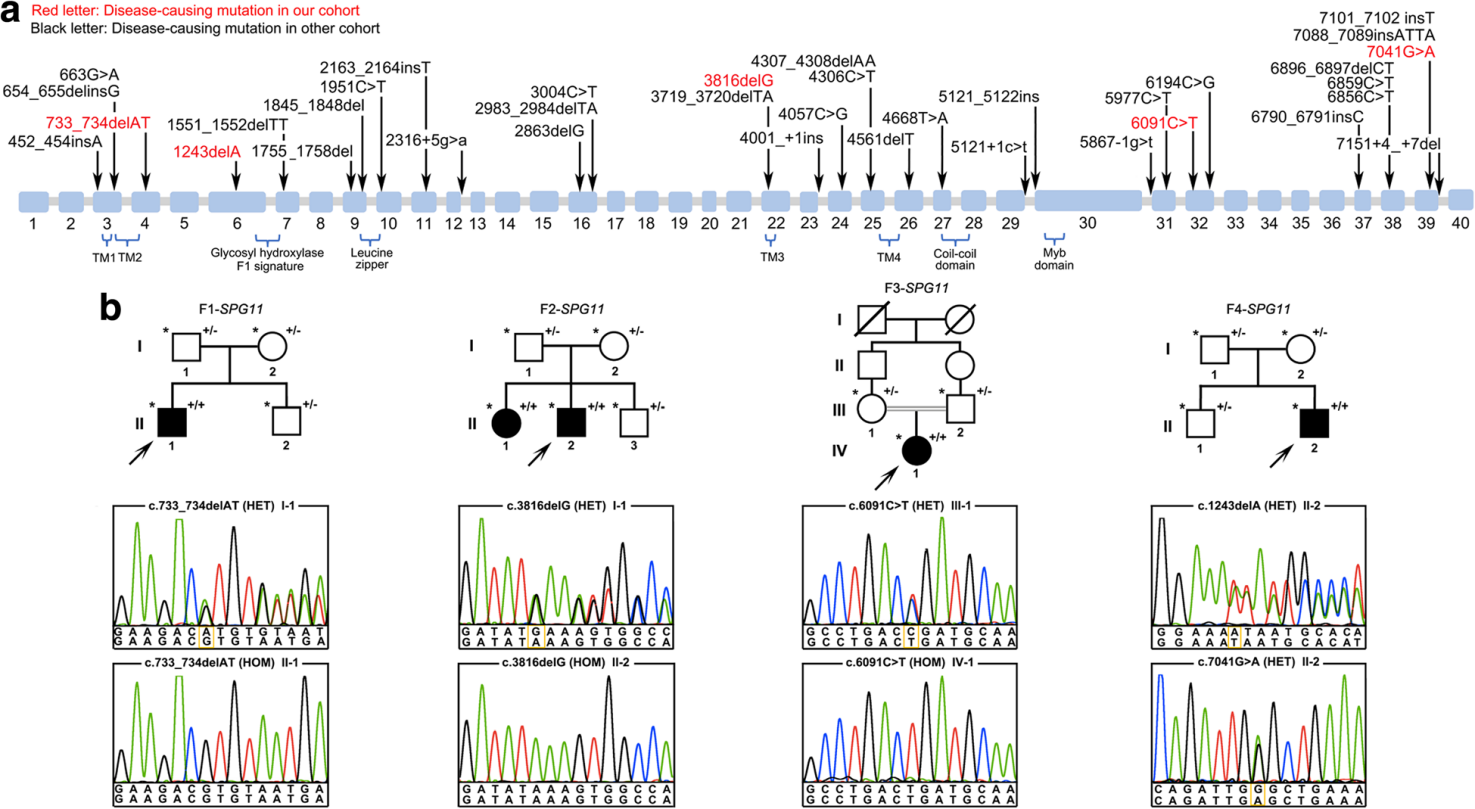
Mutational spectrum of *SPG11* gene in Chinese SPG11 patients. **a** Schematic representation of the mutational location in cDNA of *SPG11* gene detected in Chinese SPG11 patients (*n* = 52). Mutations detected in our cohort are indicated in red characters. Four transmembrane domains (TM1, TM2, TM3 and TM4), Glycosyl hydroxylase F1 signature, Leucine zipper, Coil-coil domain and Myb domain are highlighted. **b** Pedigree chromatograms and sanger sequencing of four SPG11 families in our cohort. Three probands carried homozygous LOF mutations: c.733_734delAT (p.M245Vfs^*^2) in family F1, c.3816delG (p.M1272Ifs^*^6) in family F2 and c.6091C > T (p.R2031^*^) in family F3. The index case in family F4 carried compound heterozygous LOF mutations: c.1243delA (p.I415^*^) (I-2) and **c** 7041G (p.W2234^*^) (I-1). Symbol with “+/+” indicate patient. Symbol with “+/−” indicate mutation carrier; Symbol with “*” indicate the member whose sample was available. Mutations were marked by yellow box

A total of 31 subtle mutations have been detected in Chinese SPG11 patients so far (Fig. 4a). In our cohort, we identified five LOF mutations (p.M245Vfs^*^2, p.I415^*^, p.M1272Ifs^*^6, p.R2031^*^ and p.W2234^*^) of *SPG11* gene in 4 SPG11 families (Fig. 4b). Sanger sequencing of these mutations in other family members showed a co-segregation of genotype with the disease phenotype. However, we did not find obvious clustering of these 36 mutations on the functional domain of spatacsin protein, encoded by *SPG11* gene (Fig. 4a).

#### Mutations in *CYP7B1* gene cause ARHSP

SPG5 is caused by mutations in *CYP7B1* gene [19]. The mean ± SD AAO in Chinese SPG5 patients was 16.88 ± 10.39 years, ranging from 3 to 44 years old, with a peak in the first two decades. The mean ± SD disease duration was 9.93 ± 7.17 years. More than half of the patients (57.1%) presented as complicated forms. Interestingly, white matter lesions were observed in 50% of complicated SPG5 patients (Additional file 1: Table S7).

In our cohort, mutations in *CYP7B1* gene was identified in 16 SPG5 kindred (Additional file 2: Figure S4). Among these, 14 families carried the known nonsense mutation c.334 C > T (p.R112^*^) [20], which was homozygous in 13 families, accounting for up to 92.9%. Haplotype analysis was further performed on members of the 16 unrelated families, and revealed that cases with the mutation c.334C > T (p.R112^*^) shared the same unique haplotype in the 1.5-Mb interval between tagSNP rs9298109 and rs4367588 (Additional file 1: Table S8). This result implied that all the specific SPG5 patients carrying the homozygous mutation c.334C > T (p.R112*), originated from the common ancestor, which is termed as ‘founder effect’ (Additional file 1: Table S8). Besides that, we also identified another four novel mutations (p.L145Cfs^*^13, p.Y181^*^, p.R370C and p.P397L) of *CYP7B1* in another 3 remaining families.

#### Mutations in *DDHD1*, *FA2H*, *C19orf12, GBA2*, and *DDHD2* gene cause ARHSP

Mutations in *DDHD1* (SPG28), *C19orf12* (SPG43) and *DDHD2* (SPG54) gene of our ARHSP patients were firstly presented in China. One previously mutation (p.G58R in *C19orf12)* and three novel mutations (p.R740^*^ in *DDHD1*, p.Y99^*^ and p.R112Q in *DDHD2*) were identified in three ARHSP families (Additional file 2: Figure S5). All these three families mainly presented with weakness and spasticity restricted to the lower limbs, thus they were considered as a pure form of HSP. In addition, we have also confirmed four novel mutations (p.P148L and p.L131Sfs^*^14 in *FA2H* (SPG35), p.W475^*^ and p.R879W in *GBA2* (SPG46)) in two ARHSP families (Additional file 2: Figure S5)*.* Both of them showed additional complicated features including dysarthria, cognition impairment, even skin peeling.

### Functional characterization of mutant spastin and spatacsin in the patients derived fibroblast cells

SPG4 and SPG11 are the most common subtypes of ADHSP and ARHSP in China, respectively. The two groups account for up to 68.5% of all patients. To elucidate their pathogenic mechanism, we further study the function of spastin and spatacsin protein in patients derived fibroblasts.

#### Loss of spastin results in abnormal mitochondrial dynamics and oxidative metabolism

**Fig. 5:**
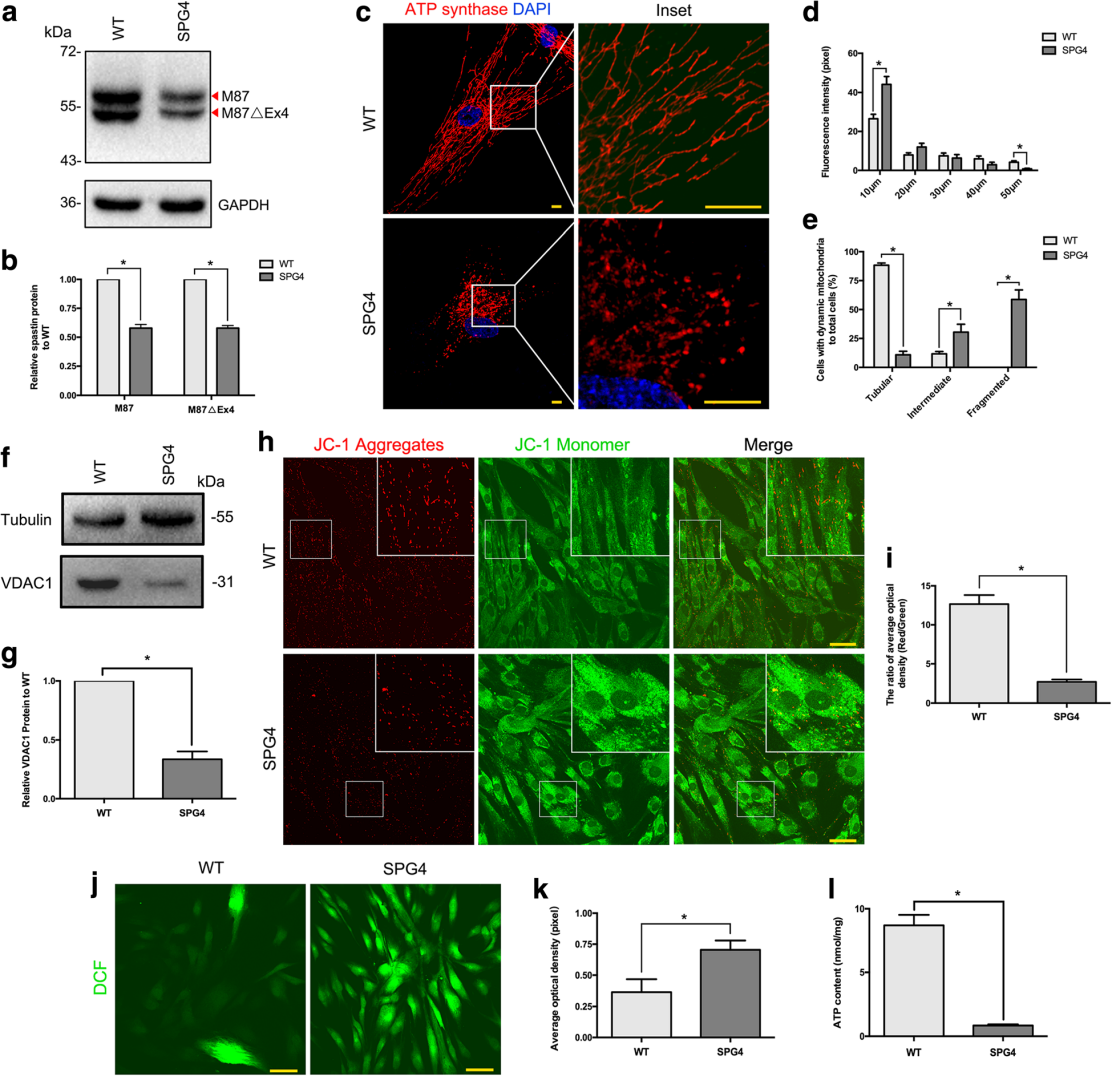
Mitochondrial dynamics and oxidative metabolism were altered in the SPG4 patient derived fibroblasts. **a**, **b** Western blot analysis showed a significant difference in spastin isoforms (M87 and M87△Ex4) expression levels between the SPG4 patient and WT. **c** ICH of mitochondria maker (ATP synthase, red) and DAPI (blue) in fibroblasts from the WT (upper panel) and the SPG4 patient (lower panel). Insets in the images are enlarged (original magnification, ×8.0) to the right. Scale bar, 10 μm. **d** Quantification of mitochondria distribution measured from the center of the cells (*n* = 3, > 10 cells per experiment). The data showed that the number of mitochondria were significantly reduced along the branch in the SPG4 patient derived fibroblasts. **e** Quantification of cells with different mitochondrial morphology (tubular, intermediate and fragmented patterns) to total cell (*n* = 3, > 30 cells per experiment). **f**, **g** Western blot analysis showed a significant difference in VDAC1 protein expression levels between the SPG4 patient and WT. **h** JC-1 staining was used to measure MMP (Δψm) in fibroblasts from the WT (upper panel) and the SPG4 patient (lower panel). Boxed areas are enlarged in the insets. Scale bar, 50 μm. **i** Statistical analysis of h (*n* = 3). **j** Intracellular ROS visualization using DCFH-DA staining in WT (upper panel) and SPG4 (lower panel). Scale bar, 50 μm. **k** Statistical analysis of (**j**) (*n* = 3). **l** ATP bioluminometers analysis revealed a significant decrease of ATP content in the SPG4 patient. Error bars indicate SEM. *, *P* < 0.05

In fact, there are two additional spastin isoforms, M1△Ex4 and M87△Ex4, that result from skipping of exon 4. We observed that the M87 and M87△Ex4 isoforms were both present in both WT and SPG4 fibroblasts, but not with M1 or M1 △Ex4 isoforms (Fig. 5a). The level of both two predominant isoforms were significantly reduced by 43% in SPG4 (*P* < 0.001, Fig. 5b). The premature termination codon within the AAA cassette domain of spastin is expected to induce nonsense mediated mRNA decay [21]. Consistent with this, we did not find the truncated isoforms (~ 50 kDa) in the SPG4 fibroblasts (Fig. 5a).

**Fig. 6:**
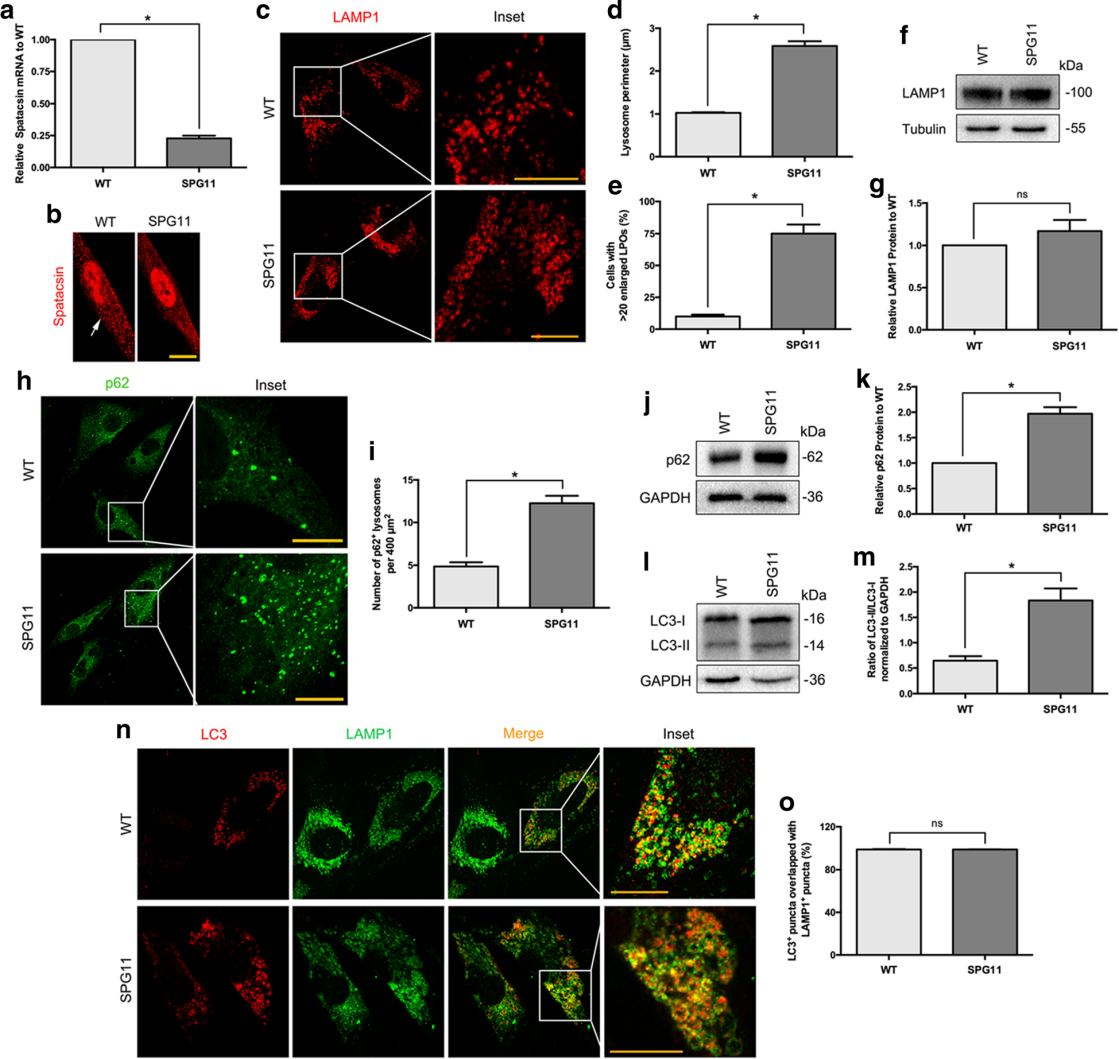
Fibroblasts from the SPG11 patient exhibit enlarged LAMP1-positive organelles and accumulation of autolysosomes. **a** QPCR analysis revealed a significantly decreased expression of spatacsin mRNA in the pateint’s fibroblasts. **b** ICH of spatacsin (red particles, arrow indicated) in fibroblast of WT (left panel) and the SPG4 patient (right panel). **c** ICH analysis revealed fibroblasts from the SPG11 patient exhibit prominently enlarged LPOs. Insets in the images are enlarged (original magnification, × 9.0) to the right. **d** Quantification of lysosome perimeter (20 cells, > 20 lysosomes per cell). **e** Quantification of cells with exceed 20 enlarged LPOs (*n* = 3, > 30 cells per experiment). **f**, **g** Western blot analysis showed no significant difference in LAMP1 levels between the SPG11 patient and WT. **h** ICH of p62 (green) in fibroblast of WT (upper panel) and the SPG11 patient (lower panel). Insets in the images are enlarged (original magnification, ×9.0) to the right. **i** Quantification of h (50 cells). **j**, **k** Western blot analysis revealed p62 protein was significantly accumulated in the patient derived fibroblasts. **l**, **m** Western blot analysis showed the ratio of LC3-II/LC3-I was significantly increased in autophagy induced fibroblasts from the SPG11 patient. **n**, **o** Co-immunostaining with LC3 (red) and LAMP1 (green), which showed no significant difference of their overlapping between the two groups. Insets in the images are enlarged (original magnification, ×10.5) to the right. Scale bar, 10 μm. Error bars indicate SEM. *, *P* < 0.05. ns, not significant

The spastin expression levels are the limiting factor in microtubule severing to maintain microtubule network. Mitochondrial motility and regional distribution have been reported to closely interact with cytoskeletal tracks, such as microtubules and actin filaments [22]. To evaluate the mitochondrial distribution pattern in SPG4, mitochondria were visualized using antibody against ATP synthase. That we observed a more pronounced concentration of mitochondria around the perinuclear region in patient derived fibroblasts (*P* < 0.001, Fig. 5c and d), suggesting the intracellular trafficking alteration. Intriguingly, we further found more fragmented and unbranched, but fewer tubular mitochondria in SPG4 fibroblasts (*P* < 0.001, Fig. 5c and e), which implied the impaired patterns of mitochondrial fission and fusion. Mitochondrial network fragmentation was further assessed by immunoblot analysis of the voltage-dependent anion channel 1(VDAC1), an outer mitochondrial membrane protein, acts as a gatekeeper for the transport of mitochondrial metabolites [23, 24]. Likewise decreased expression of the VDAC1 protein was observed in SPG4 fibroblasts (~ 70% reduction, *P* < 0.001, Fig. 6f and g). We further sought to determine whether the mitochondrial function was altered. Fluorescence indicator, JC-1, was used to validate the level of MMP (Δψm). We observed the ratio of average optical density (AOD, red to green) was significantly reduced by 80% in SPG4 (*P* < 0.001, Fig. 6h and i). The loss of Δψm could induce the increase in intracellular ROS [25], which was strongly confirmed by DCFH-DA staining. We found that the ROS level was dramatically increased in SPG4 fibroblasts (~ 2 folds increase on average, *P* < 0.001, Fig. 6j and k). We further investigated the effect on ATP content. ATP bioluminometers analysis revealed that the relative ATP content was apparently decreased in the SPG4 fibroblasts (mean ± SEM, WT:8.70 ± 0.83 nmol/mg, SPG4: 0.85 ± 0.09 nmol/mg, *P* < 0.001, Fig. 6l). Thus, the lack of spastin in SPG4 would cause abnormal mitochondrial dynamics and oxidative metabolism.

#### Loss of spatacsin results in enlarged LAMP1-positive organelles and accumulation of autolysosomes

QPCR and ICH results showed that spatacsin expression level was significantly decreased by 80% in SPG11 fibroblasts (*P* < 0.001, Fig. 6a and b). Spatacsin has been reported to play a pivotal role in autophagic lysosome reformation, a pathway that generates new lysosomes [26]. With inducing autophagy by starvation of fibroblasts, we found SPG11 fibroblast cells exhibited prominently enlarged organelles immunostained with LAMP1 (~ 2.5 folds increase on average, *P* < 0.001), a marker of late endosomes and lysosomes (Fig. 6c and d). Moreover, the number of cells with enlarged LAMP1-positive organelles (LPOs) was also significantly increased in SPG11 (~ 8 folds increase on average, *P* < 0.001, Fig. 6e), although this had no significant effect on total LAMP1 protein level (*P* = 0.235, Fig. 6f and g). Thus, we speculated that depletion of spatacsin would result in the enlarged volume of LPOs, but not in their change of total amount.

Autophagosomes fuse with lysosomes to generate autolysosomes, which is essential for autophay-dependent intracellular clearance. The accumulation of enlarged LPOs reminds us to study any dysfunction in autophagosomes status. Indeed, SPG11 fibroblasts present significantly aggregated mature autophagosomes labeled by p62 (*P* < 0.001, Fig. 6h and i), and with enhanced p62 protein level (~ 2.0 folds increase on average, *P* < 0.001, Fig. 6j and k). During autophagy, cytosolic LC3 (LC3-I) is continually modified to form a LC3-phosphatidylethanolamine conjugate (LC3-II), which can be recruited to autophagosomal membranes. The ratio of LC3-II to LC3-I is recognized as a marker for monitoring autophagosomes formation [27]. To further validate above finding, we performed immunoblot on LC3 and confirmed a significant increase in the amount of autophagosomes in SPG11 fibroblasts (~ 3.0 folds increase on average, *P* < 0.001, Fig. 6l and m). To investigate the spatial relationship between enlarged LPOs and accumulated autophagosomes, fibroblasts were coimmunostained with LC3 and LAMP1. We found that most autophagosomes labeled by LC3 were also positive with the enlarged LPOs labeled by LAMP1 in both groups (*P* = 0.858, Fig. 6n and o), indicating that normal fusion of autophagosomes with lysosomes had occurred in spatacsin-deficiency fibroblasts. Overall, the lack of spatacsin in SPG11 would lead to aberrant accumulation of the autolysosomes.

## Discussion

Herein, we have analyzed and summarized 430 of ADHSP patients and 101 of ARHSP patients in China, respectively. In total, 18 SPG subtypes and more than 150 mutations have been uncovered. To the best of our knowledge, this study included the largest HSP cohort in China and presented a comprehensive overview of the clinical spectrum and genetic landscape for HSP.

As in previous studies, AD forms of HSP are mainly pure forms [28]. The AAO had a wide range from infancy to late adulthood [29, 30]. Mutations in *SPAST* (SPG4), *ATL1* (SPG3A), *KIF5A* (SPG10) and *REEP1* (SPG31) are reported as being responsible for more than 50% of all cases [6]. The present study has confirmed that SPG4 is the most common subtype of Chinese ADHSP patients, and followed by SPG3A, SPG6 (*NIPA1*) and SPG33 (*ZFYVE27*). Clinically, 94% of these patients were of pure HSP. However, no mutations were identified in SPG10, which appeared to be a common cause of ADHSP in previous series of other populations [31, 32]. Moreover, SPG8 (*KIAA0196*), SPG30 (*KIF1A*), SPG31and SPG42 (*SLC33A1*) seemed to be quite rare subtypes of Chinese ADHSP patients.

About 10–40% of HSP patients harbored subtle mutations and exonic rearrangements in the *SPAST* gene [3, 33]. Subtle mutations were confirmed to be the most common genetic cause for Chinese SPG4 patients, and most of them located in AAA casseete domain of Spastin protein. Spastin, a well-known AAA ATPase, is widely expressed in vertebrate cells that sever microtubules [34]. M87 is the much more abundant and effective severing isoform of spastin than M1 [35]. M1 is exclusively presented in adult spinal cord, the location of corticospinal tract axons that degenerate during HSP [21, 35]. The dynamic microtubule is functionally important for the intracellular organelles trafficking [14, 36]. Within neurons, only the short microtubules, but not the long ones, are critical for efficient axonal transport [37, 38]. In our cohort, all novel *SPAST* mutations have been shown to downregulate the expression or stability of M1- and M87-spastin protein. Based on SPG4 patient derived fibroblasts, we observed only reduced M87 and M87△Ex4 isoforms, and that mitochondrial dynamics and network were both altered. Of note, we further found the decreased MMP (Δψm), increased ROS and reduced ATP content in SPG4 fibroblasts, which have been implicated in cellular injury [39]. The neurite swelling and imbalanced axonal transport have been reported in the SPG4 stem cells derived neurons [21, 40, 41]. Indeed, in postmortem SPG4 patient brain samples, abnormal mitochondria readily accumulated in the cytosol of affected neurons [42]. On these basis, we speculate that the mitochondrial-mediated pathway plays a key role in neuron degeneration in the pathogenesis of SPG4.

In contrary to ADHSP, the AR forms of HSP appear to be more complex in clinical terms, with an early onset of symptoms [6, 43]. SPG11 was the most common cause of ARHSP [44]. In this study, 78% of Chinese ARHSP patients were suggestive of complicated HSP. Mutations in *SPG11* (SPG11) are responsioble for around 53% of ARHSP cases, and followed by *CYP7B1* (SPG5), *FA2H* (SPG35) and *GBA2* (SPG46). However, no mutations were identified in *SPG7* (SPG7) and *ZFYVE26* (SPG15) gene, which appeared to be a common cause of ARHSP in previous series of other populations [2, 6, 9, 10, 45, 46]. Indeed, subtypes for SPG14, SPG18, SPG28, SPG43, SPG54 and SPG77 of Chinses ARHSP patients are apparently orphan, affecting single family members.

In the present study, the AAO of SPG11 was generally early. The cognitive deficit, dysarthria, mental retardation, thin corpus callosum and axonal peripheral neuropathy were highly suggestive of SPG11. In previous reports, mutations in *CYP7B1* were found in both pure and complicated forms of SPG5 [47]. Cerebellar ataxia has been the most common SPG5-related complicated phenotype [48]. Of note, white matter lesions and spastic ataxia seemed to be the two major features in Chinese SPG5 cases, accounting for approximately 50%. It was of interest to note that 82.1% of these SPG5 patients carried the unique nonsense mutation c.334C > T (p.R112^*^)*.* Haplotype analysis revealed that the hotspot mutation is correlated with the founder effect.

Point mutations and rearrangements in *SPG11* have been show to make up 20% of ARHSP [49]. In our cohort, all five mutations of *SPG11* were stopgain mutation, with predicting to be LOF. Emerging studies implicated that spastic paraplegia proteins, spatacsin (SPG11) and spastizin (SPG15), were the essential components for the initiation of lysosomal tubulation [26, 50, 51]. Dysfunction of spatacsin leads to impaired axonal maintenance and cargo trafficking in the SPG11 stem cells derived neurons [52, 53]. Based on SPG11 patient derived fibroblasts, we observed that lack of spatacsin indeed induced the enlargement of LPOs and accumulation of autophagosomes. More importantly, the essence of these abnormally accumulated vesicles was shown to be autolysosomes, whose quantity and quality are crucial for autophagy. Autophagy is an intracellular activity for degradation and recycling of cytoplasmic components to maintain homeostasis [54]. The perturbed autophagy has been linked to several neurodegeneration diseases [55]. Collectively, our findings implied that the presence of autophagic dysfunction is tightly linked to SPG11.

## Conclusions

In summary, our results indicated that subtypes for SPG4, SPG3A, SPG6 and SPG33 were the most frequently found in Chinese ADHSP patients, and were mainly pure forms with wide range AAO. Meanwhile, SPG11 SPG5, SPG35 and SPG46 subtypes were the most frequently found in Chinese ARHSP patients, and were more complex in clinical terms with early AAO. Moreover, we further determined that the high frequency of mutations in *CYP7B1* gene resulted from the founder effect. These studies provide the important implications on genetic counseling for suspected HSP cases. All of included patients presented with a wide range of features from asymptom to multi-system involvement, which confirming the marked heterogeneity of HSP. Stressing the mitochondrial and autophagic dysfunction hold promise as a potential therapeutic target to halt the progression of neurodegeneration in HSP. Even though, further study of a large cohort of patients should ascertain the specific correlation of genotype-phenotype in each subtype of HSP.

## Additional files

Additional file 1: Summary information for mutational spectrum, targeted sequencing genes, amplification primers, antibodies, clinical features and haplotype analysis. **Table S1**. A reanalysis of the mutational spectrum in 41 reported studies exhibiting HSP in China. **Table S2**. HSP related genes included in the targeted sequencing panel. **Table S3**. Primers for amplification in the experimental procedures. **Table S4**. List of antibodies, related to immunocytochemistry (ICH) and western blotting (WB) in the experimental procedures. **Table S5**. Summary of clinical features of SPG4 patients in China (*n*=273). **Table S6**. Summary of clinical features of SPG11 patients in China (*n*=52). **Table S7**. Summary of clinical features of SPG5 patients in China (*n*=28). **Table S8**. Haplotypes of tagSNPs linkage to *CYP7B1* gene in the 16 unrelated SPG5 families. (XLSX 69 kb)

Additional file 2: Pedigrees, sequencing chromatograms of disease-causing gene related to HSP families in our cohort. **Figure S1**. Pedigree, sequencing chromatograms of *SPAST* gene detected in 16 SPG4 families in our cohort. **Figure S2**. Western blot analysis of novel mutations of *SPAST* gene in HEK 293T cells. **Figure S3**. Pedigree, sequencing chromatograms of 6 ADHSP families in our cohort. **Figure S4**. Pedigree, sequencing chromatograms of *CYP7B1* gene detected in 16 unrelated SPG5 families in our cohort. **Figure S5**. Pedigree, sequencing chromatograms of 5 ARHSP families in our cohort. (DOCX 9157 kb)

## Abbreviations

AAA: *A*TPases *a*ssociated with diverse cellular *a*ctivities
AAO: Age at onset
AD: Autosomal dominant
ADHSP: Autosomal dominant hereditary spastic paraplegia
AR: Autosomal recessive
ARHSP: Autosomal recessive hereditary spastic paraplegia
DCFH-DA: Dichlorofluorescein diacetate
DMEM: Dulbecco’s modified Eagle’s medium
ExAC: Exome Aggregation Consortium database
FBS: Fetal bovine serum
HSP: Hereditary spastic paraplegias
ICH: Immunocytochemistry
LOF: Loss-of-function
LPOs: LAMP1-positive organelles
MIT: Microtubule interacting and trafficking
MLPA: Multiplex ligation-dependent probe amplification
MMP: Mitochondrial membrane potential
MRI: Magnetic resonance images
MTBD: Microtubule-binding domain
NGS: Next generation sequencing
PCR: Polymerase chain reaction
qPCR: Quantification PCR
ROS: Reactive oxygen species
RT-PCR: Reverse transcription polymerase chain reaction
SPG: Spastic paraplegia
TM: Transmembrane
VDAC: Voltage-dependent anion channel
