# Supplementary material for: Clinical spectrum and genetic landscape for hereditary spastic paraplegias in China
Source: Mol Neurodegener. 2018 Jul 6;13:36. doi: 10.1186/s13024-018-0269-1 (PMC6035405; doi:10.1186/s13024-018-0269-1)
Supplement: Supplementary file 2 — Pedigrees, sequencing chromatograms of disease-causing gene related to HSP families in our cohort. Figure S1. Pedigree, sequencing chromatograms of SPAST gene detected in 16 SPG4 families in our cohort. Figure S2. Western blot analysis of novel mutations of SPAST gene in HEK 293T cells. Figure S3. Pedigree, sequencing chromatograms of 6 ADHSP families in our cohort. Figure S4. Pedigree, sequencing chromatograms of CYP7B1 gene detected in 16 unrelated SPG5 families in our cohort. Figure S5. Pedigree, sequencing chromatograms of 5 ARHSP families in our cohort. (DOCX 9157 kb) [file 13024_2018_269_MOESM2_ESM.docx]

Supplementary Figure 1. Pedigree, sequencing chromatograms of *SPAST* gene detected in 16 SPG4 families in our cohort.


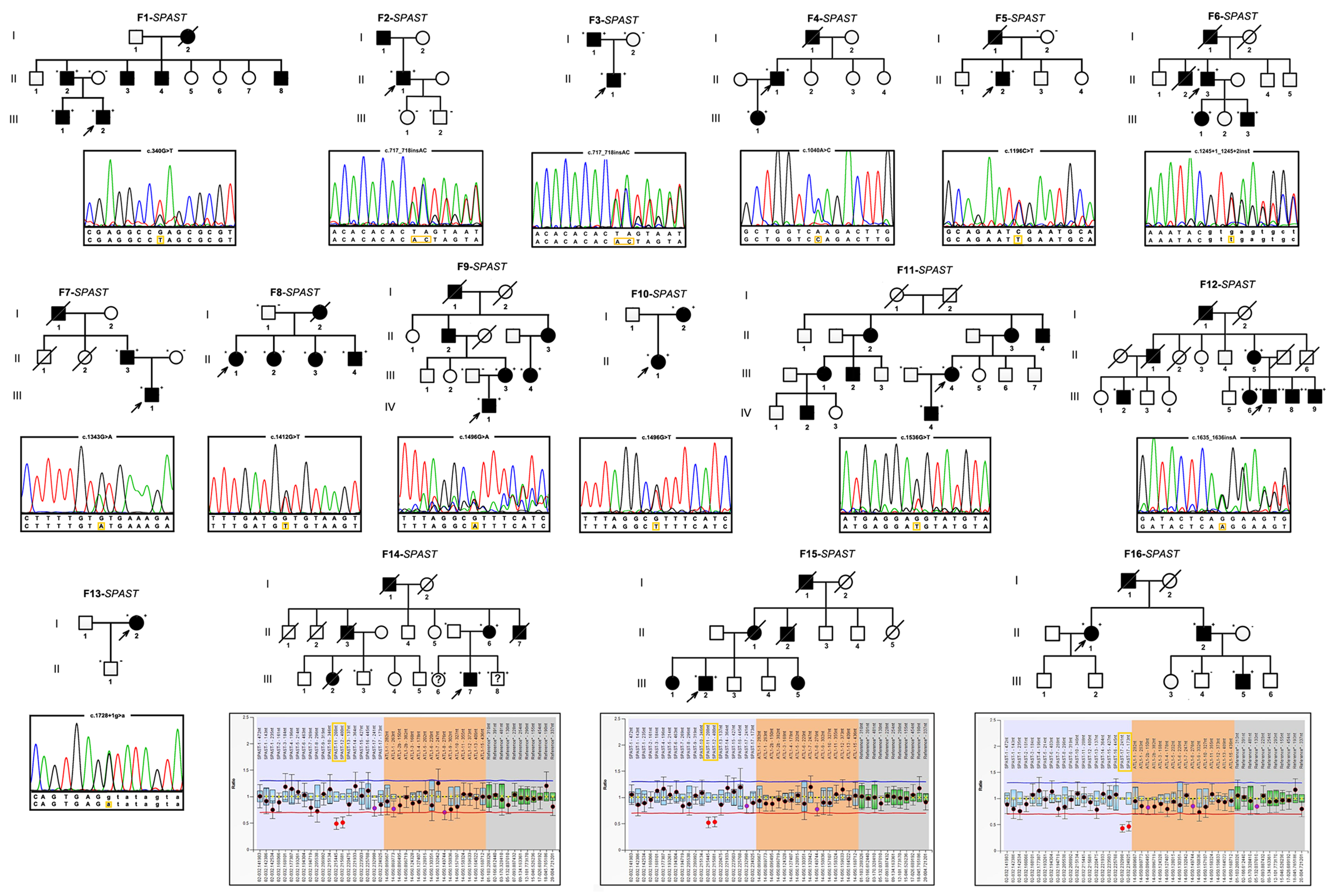


Supplementary Figure 2. Western blot analysis of novel mutations of *SPAST* gene in HEK 293T cells.


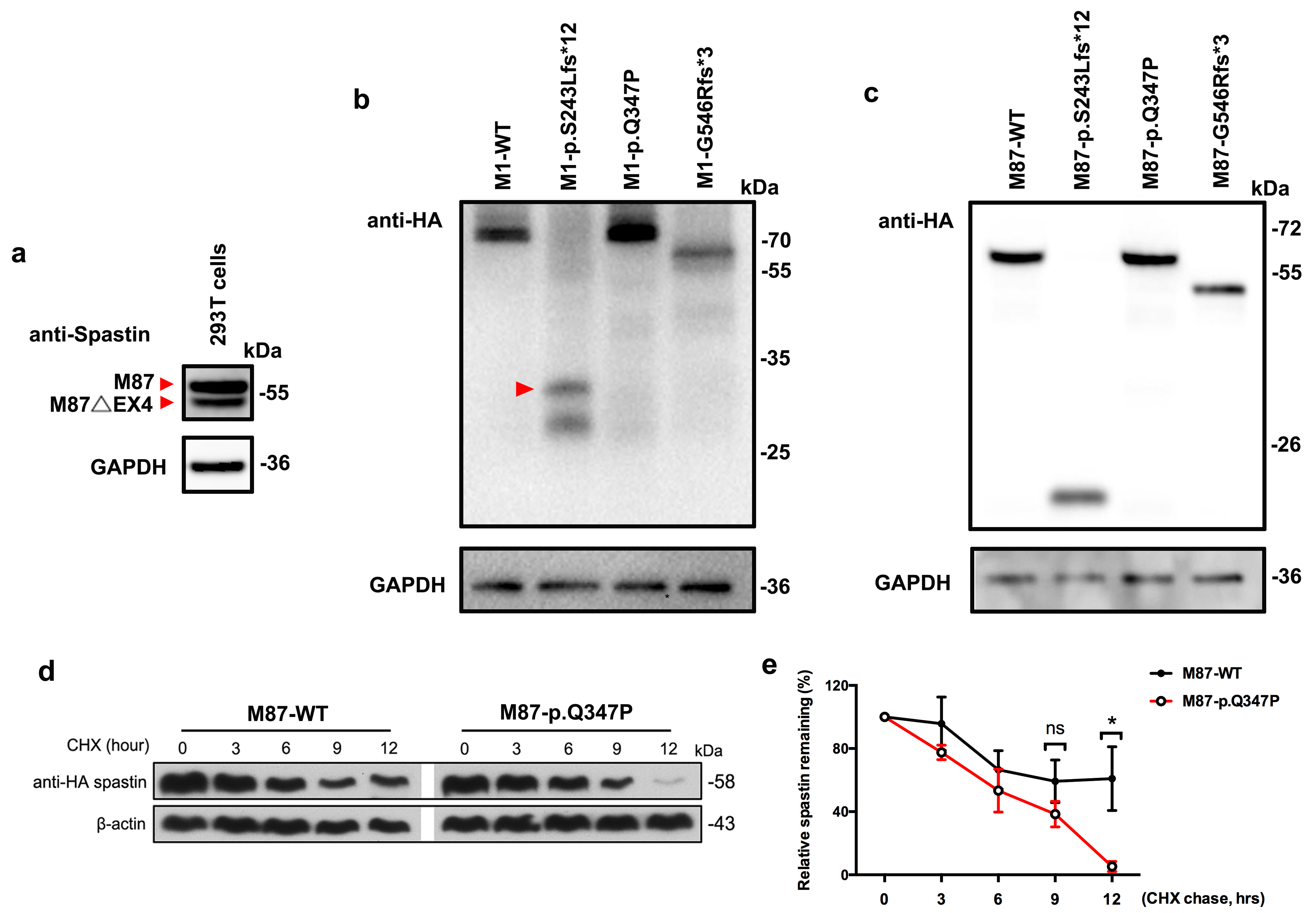


Supplementary Figure 3. Pedigree, sequencing chromatograms of 6 ADHSP families in our cohort.


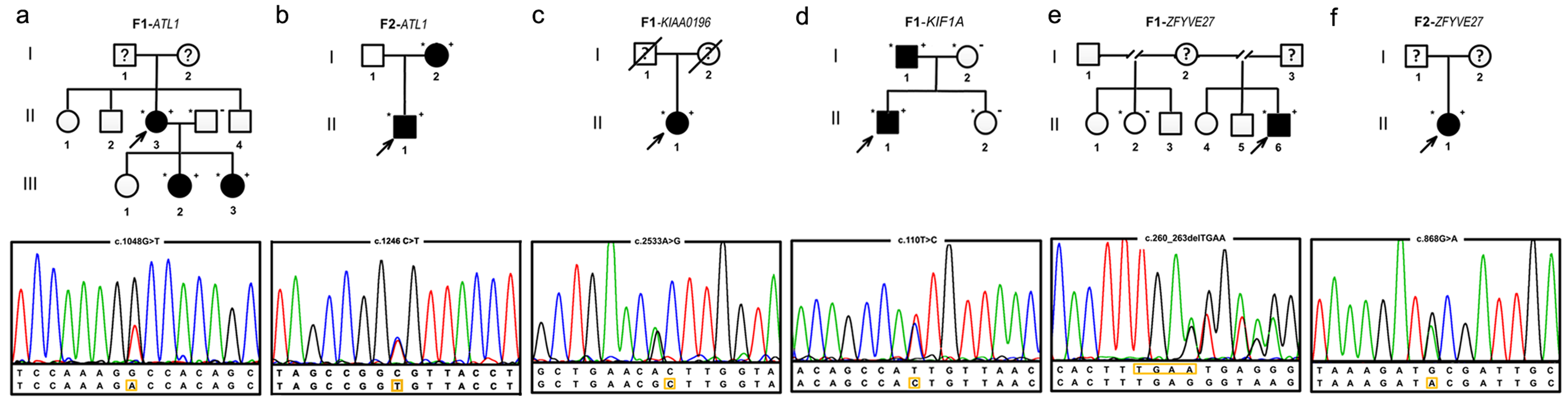


Supplementary Figure 4. Pedigree, sequencing chromatograms of *CYP7B1* gene detected in 16 unrelated SPG5 families in our cohort.


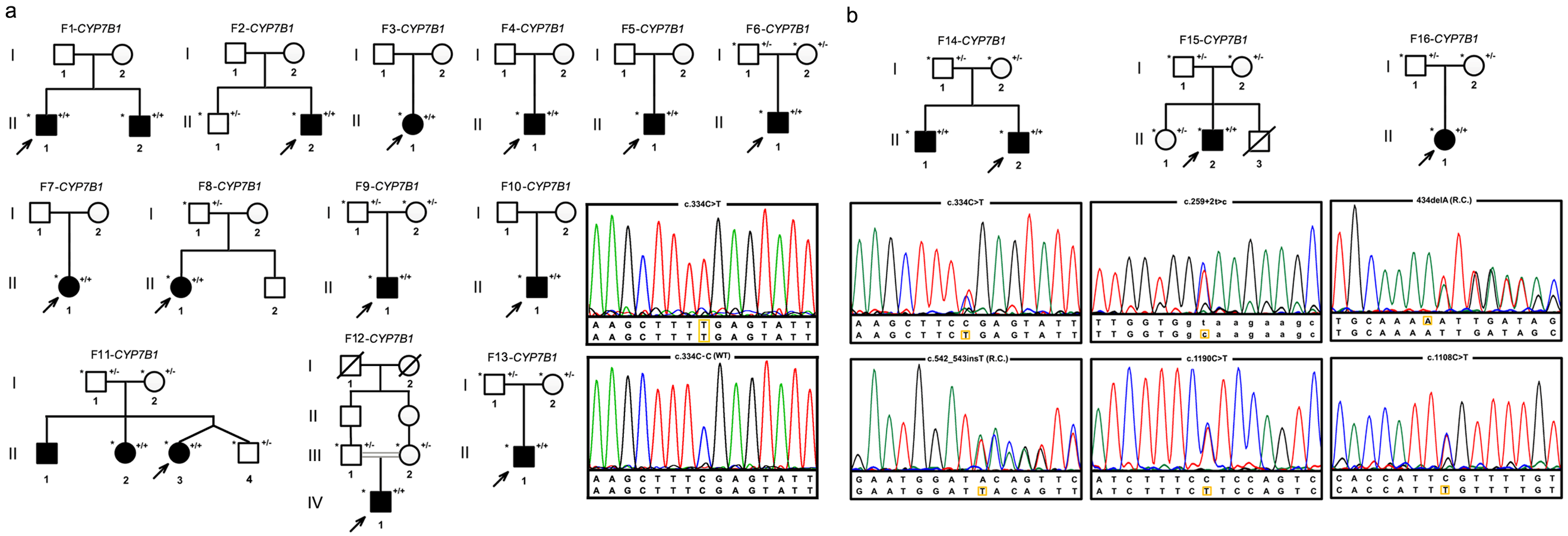


Supplementary Figure 5. Pedigree, sequencing chromatograms of 5 ARHSP families in our cohort.


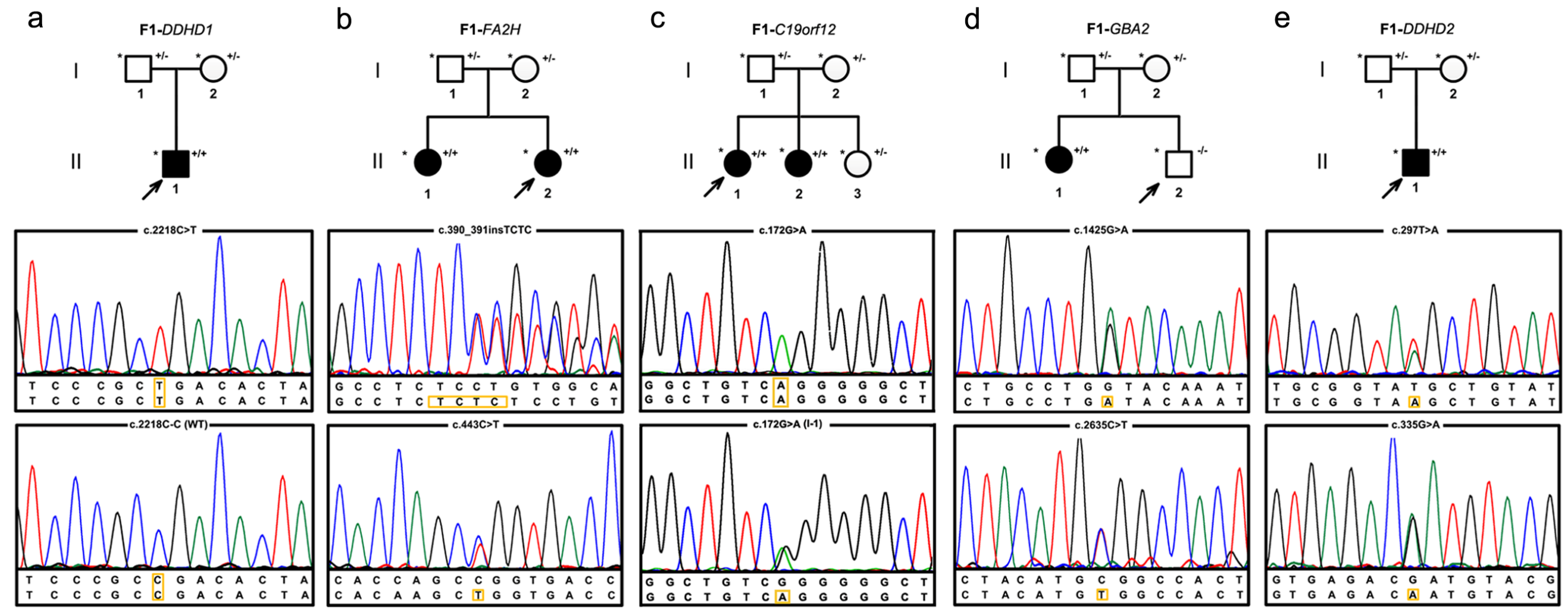


**Supplementary Figure legends**

**Fig. S1. Pedigree, sequencing chromatograms of *SPAST* gene detected in 16 SPG4 families in our cohort.** The probands of family F1-F13 carried micro mutations, and the index cases of family F14-F16 harbored exon deletion mutations. Mutations detected in *SPAST* gene were marked by yellow box. Symbols with a “+” indicate patients. Symbols with a “-” indicate healthy control. Symbols with a “*” indicate members whose sample was available.

**Fig. S2. Western blot analysis of novel mutations of *SPAST* gene in HEK 293T cells.** a. The spastin isoforms of HEK293T cells were probed with antibody against its endogenous spastin protein. Immunoblot results revealed that M87 and M87△Ex4 isoforms are the predominant bands in 293T cells, but not with M1 or M1△Ex4 isoforms. b, c. To distinguish from the endogenous spastin in HEK293T cells, the exogenous overexpressed spastin protein were probed with anti HA flag antibody. Immunoblot results showed that only the M1 (b) and M87 (c) isoforms of exogenous spastin was detected, but not with M1△Ex4 or M87△Ex4 isoforms. Two stopgain mutations p.S243Lfs^*^12 and p.G546Rfs^*^3 induced the truncation of spastin, whereas missense mutation p.Q437P did not affect spastin protein expression. Red triangle indicates the target band. d. Time-course stability analysis of mutant spastin (M87-p.Q347P) by western blot . Cells were collected at 0, 3, 6, 9, 12 h following treatment with cycloheximide (40 μg/ml). e. Statistical analysis of d (n=3). CHX treatment led to spastin protein degradation in a time-dependent manner. Error bars indicate SEM. *, *P*<0.05. ns, not significant.

**Fig. S3. Supplementary Figure 3. Pedigree, sequencing chromatograms of 6 ADHSP families in our cohort.** Two SPG3A families (*ATL1* gene, a, b), one SPG8 family (*KIAA0196* gene, c), one SPG30 family (*KIF1A* gene, d) and two SPG33 families (*ZFYVE27* gene, e, f). Mutations were marked by yellow box. Symbols with a “+” indicate patients. Symbols with a “-” indicate healthy control. Symbols with a “*” indicate members whose sample was available.

**Fig. S4**. **Pedigree, sequencing chromatograms of *CYP7B1* gene detected in 16 unrelated SPG5 families in our cohort.** The probands of family F1-F13 carried homozygous mutation c.334C>T (p.R112^*^) (a), and the index cases of family F14-F16 harbored compound heterozygous mutations (b): c.334C>T (I-1) and c.542_543insT (I-2) in family F14, c.259+2t>c (I-2) and c.1190C>T (I-1) in family F15, c.434delT (I-1) and c.1108C>T (I-2) in family F16. Mutations were marked by yellow box. Symbols with a “+/+” indicate patients. Symbols with a “+/-” indicate carrier. Symbols with a “*” indicate members whose sample was available. R.C., reverse plus complement.

**Fig. S5**. **Pedigree, sequencing chromatograms of 5 ARHSP families in our cohort.** The probands of SPG28 and SPG43 families carried homozygous mutation: c.2218C>T (*DDHD1* gene, a) and c.172G>A (*C19orf12* gene, c), and the index cases of SPG35, SPG46 and SPG54 families harbored compound heterozygous mutations: c.390_391insTCTC (I-1) and c.443C>T (I-2) in *FA2H* gene (b), c.1425G>A (I-2) and c.2635C>T (I-1) in *GBA2* gene (d), c.297T>A (I-2) and c.335T>A (I-1) in *DDHD2* gene (e). Mutations were marked by yellow box. Symbols with a “+/+” indicate patients. Symbols with a “+/-” indicate carrier. Symbols with a “*” indicate members whose sample was available.
